# Temporal dynamics of ICP, PRx, CPP, and CPPopt in relation to functional outcome in spontaneous cerebellar hemorrhage

**DOI:** 10.1186/s13054-026-06136-0

**Published:** 2026-06-10

**Authors:** Rozerin Kevci, Anders Hånell, Hilin Sida, Modar Alhamdan, Fartein Velle, Anders Lewén, Andreas Fahlström, Per Enblad, Teodor Svedung Wettervik

**Affiliations:** Department of Medical Sciences, Section of Neurosurgery, Uppsala University, Uppsala, Sweden

**Keywords:** Cerebral pressure autoregulation, Cerebral perfusion pressure, Intracranial pressure, Neurointensive care, Outcome, Spontaneous cerebellar hemorrhage

## Abstract

**Background:**

Spontaneous cerebellar hemorrhage (sCH) is a severe condition, due to the limited space in the posterior fossa. The temporal dynamics of cerebral physiological variables such as intracranial pressure (ICP), pressure reactivity index (PRx), cerebral perfusion pressure (CPP), “optimal” CPP (CPPopt), and CPP deviation from CPPopt (ΔCPPopt) and associations with functional outcome remain insufficiently characterized in sCH.

**Methods:**

This study retrospectively analyzed 94 adult sCH patients treated at the neurointensive care (NIC) unit in Uppsala University Hospital, Sweden, between 2008 and 2024, with collected high resolution monitoring data. The association between insult intensity-duration, temporal dynamics, and interactions with cerebrovascular autoregulation of the cerebral physiological variables with Glasgow Outcome Scale at discharge (GODS) were analyzed in outcome heatmaps. Formal statistical analyses between % of good monitoring time (%GMT) of the cerebral physiological variables within/outside certain thresholds were analyzed in relation to GODS.

**Results:**

In the exploratory analyses, ICP > 15 mmHg demonstrated a descriptive trend toward unfavorable outcome irrespective of duration, particularly the first two days post-injury. Still, the association lacked significance (*p* = 0.85). Sustained PRx elevations > 0.2 showed a descriptive trend toward worse outcome, with expanding vulnerability over time. However, this association was not significant (*p* = 0.26). CPP < 80 mmHg was associated with worse outcome, particularly if PRx was elevated. In addition, CPP < 60 mmHg was associated with worse outcome, while CPP > 80 mmHg was associated with favorable outcome (*p* = 0.02, respectively). Negative ΔCPPopt for longer durations and if PRx was elevated correlated with lower GODS. ΔCPPopt < -5 mmHg was associated with worse outcome (*p* = 0.04).

**Conclusions:**

Cerebral physiological monitoring provides important prognostic information in sCH. Optimal targets differ from supratentorial acute brain injuries, with an apparent benefit of higher CPP in sCH. ICP, CPP, and ΔCPPopt remain central, warranting larger multi-center studies to define sCH-specific targets.

**Supplementary Information:**

The online version contains supplementary material available at 10.1186/s13054-026-06136-0.

## Introduction

Spontaneous cerebellar hemorrhage (sCH) is a subtype of intracranial bleeding and represents approximately 5–10% of intracerebral hemorrhages [1]. It is associated with a particularly high risk of rapid neurological deterioration and mortality if untreated, owing to the space-restricted anatomical compartment of the posterior fossa, where even modest volume increases may rapidly result in mass effect, brainstem compression, and obstructive hydrocephalus [1–3]. Functional outcome in sCH is therefore highly dependent on prompt surgical intervention, when indicated, and timely neurointensive care (NIC). Regarding NIC management, intracranial pressure (ICP) monitoring constitutes a cornerstone, as elevated ICP accompanied by reduced cerebral perfusion pressure (CPP) are well-recognized causes of secondary brain injury [4–6]. Notably, differences in pressures in the supra- and infratentorial compartments have been described earlier and may be of great relevance for what constitutes harmful ICP elevations of insufficient CPP [7–9]. Several studies have reported significant differences in ICP values depending on both lesion location and ICP monitoring [7, 10, 11]. Despite this, standardized clinical management of infratentorial mass lesions commonly relies on supratentorial ICP measurement [12–14], as the clinical experience of infratentorial ICP monitoring remains limited. Additionally, supratentorial ICP monitoring remains necessary in sCH due to the risk of obstruction of the fourth ventricle causing obstructive hydrocephalus [1–3, 10, 13].

Furthermore, the cerebral pressure autoregulatory (CPA) capacity may impact the susceptibility of developing secondary brain injury. This function can be assessed using the pressure reactivity index (PRx), which is the degree of statistical correlation between slow wave components of arterial blood pressure (ABP) and ICP signals [15, 16]. From PRx, another CPA variable can be calculated – an individualized or optimal CPP (CPPopt) – defined as the CPP with the concurrently lowest PRx value over the last 4 h [17]. Studies on other acute brain injuries such as supratentorial intracerebral hemorrhage (ICH), traumatic brain injury (TBI), and subarachnoid hemorrhage (SAH) have found that impaired CPA, as indicated by a positive PRx, is associated with poor outcome [18–20]. Additionally, time spent with CPP below CPPopt has also been shown to be unfavorable in TBI [21]. However, to date, the clinical utility of CPA indices in sCH remains largely unexplored. Moreover, secondary brain injury is a dynamic process that evolves over time, and the unfavorable effects of ICP, PRx, CPP, and ΔCPPopt (CPP – CPPopt) may vary across different phases after hemorrhage [22, 23]. Notably, CPPopt is not a fixed target but shifts dynamically with changes in autoregulatory state, medical/surgical intervention, and hemodynamic conditions – a phenomenon previously described in supratentorial TBI and SAH [22–24]. Understanding these temporal dynamics is therefore essential for interpreting CPPopt-guided targets in the context of sCH. Previous studies on supratentorial ICH, TBI, and SAH have demonstrated that both the duration and timing of cerebral physiological insults are critical determinants of outcome [18, 19, 24]. Comparable high-frequency temporal analyses in sCH are lacking, despite the fact that secondary brain injury development in eloquent, penumbral regions may be detrimental. Current evidence guiding optimal cerebral physiological targets during NIC in sCH remains sparse and NIC management strategies largely extrapolate from experience in supratentorial ICH, TBI, and SAH [4, 5, 25]. Hence, their applicability to sCH is uncertain due to differences in patient characteristics, anatomy, and pathophysiology.

Altogether, there is a need to better characterize the temporal dynamics and threshold-dependent associations of ICP, PRx, CPP, and ΔCPPopt with functional outcome among sCH patients. This could help refine sCH-specific NIC targets, identify periods of increased cerebral physiological vulnerability, and determine whether CPA-guided strategies, based on the surrogate measures PRx and ΔCPPopt, offer added value over fixed physiological thresholds in patients with sCH.

## Materials and methods

### Patients and study design

This retrospective study was conducted at the Department of Neurosurgery, Uppsala University Hospital, Sweden. All patients with sCH (diagnostic codes I61.3 and I61.4) treated at the NIC or neurointermediate unit, between January 1, 2008, and August 31, 2024, were eligible. Exclusion criteria were < 12 h of total ICP monitoring during the first seven days post-injury, sCH cases due to secondary etiology (e.g., traumatic hemorrhage, intracranial tumors, vascular malformations, or neurosurgical procedures), missing clinical and radiological data, and age < 18 years. The final study comprised 94 sCH patients (Supplementary Fig. 1).

### Management protocol

The Department of Neurosurgery at Uppsala University Hospital is a tertiary referral center serving approximately two million inhabitants. Most patients with sCH were initially managed at referring hospitals according to general guidelines [5, 26, 27]. Those with moderate-to-large sCH volume on initial or follow-up computed tomography (CT) scan, limited comorbidity burden, and typically aged < 80–85 years who were deemed likely to benefit from neurosurgical care were admitted to the NIC or neurointermediate care unit.

Surgical intervention was considered in patients with Glasgow Coma Scale (GCS) ≤ 13 and radiological evidence of substantial infratentorial mass effect, particularly brainstem and/or fourth ventricle compression. An EVD was inserted into the right frontal horn if requirements were met. Additionally, in patients with smaller sCH but substantial intraventricular hemorrhage (IVH) and hydrocephalus, EVD placement alone was considered [28]. Furthermore, surgery also entailed suboccipital bony decompression (additional C1 laminectomy, if indicated), evacuation of the sCH, and often duraplasty.

NIC treatment targets were ICP < 20 mmHg, CPP > 60 mmHg, normothermia, pO_2_ > 12 kPa, arterial glucose 5–10 mmol/L, and electrolytes within ranges [28]. Unconscious patients were intubated and mechanically ventilated with normoventilation, which shifted to mild hyperventilation (pCO_2_ 4.0-4.5 kPa) in case of elevated ICP in the acute phase. Sedation and analgesia were provided with propofol and morphine, respectively. Neurological wake-up tests were performed 3–6 times daily unless ICP was elevated, in which case sedation was maintained [18]. The EVD was initially closed and opened at + 15 mmHg in case of elevated ICP or hydrocephalus, with gradual adjustments of ICP drainage level guided by ICP trends, cerebrospinal fluid (CSF) drainage volumes, and clinical and radiological responses [18]. ABP was continuously measured via radial needle at heart level. Low ABP/CPP was mainly managed with intravenous fluids and, if required, vasopressors/inotropes, while severe hypertension was treated with labetalol and/or nimodipine [18].

### Data acquisition and processing

Demography, admission, imaging, treatments, and functional outcome were obtained via systematic review of medical records and administrative databases. Hematoma volume was calculated using the ABC/2 method on the CT with the largest hemorrhage [29].

ICP data originated from the EVD, where all physiological variables were acquired at 100–125 Hz using the Odin software [30]. Artifact identification was performed using predefined physiological threshold criteria: ICP values below − 10 mmHg or above 100 mmHg, and ABP values below 0 mmHg or above 250 mmHg were excluded. PRx was calculated as a moving 5-minute Pearson correlation between 10-second averages of ABP and ICP [31, 32]. CPPopt was continuously estimated as the CPP with the lowest PRx over the previous 4-hour window [33] and was available for 57% of CPP monitoring time. ΔCPPopt was defined as the difference between actual CPP and CPPopt (ΔCPPopt = CPP_actual_ – CPPopt) [34]. All data were down-sampled to minute-by-minute values. Periods of physiological monitoring interrupted by procedures (e.g., imaging or surgery) causing artefacts such as physiologically implausible values, signal drop-outs, and periods of zero variance indicating monitoring disconnection were excluded. Hence, the remaining valid monitoring time was denoted as good monitoring time (GMT).

### Physiological analyses

Cerebral physiological analyses were conducted during the first seven days after ictus, during which median values of ICP, PRx, CPP, and CPPopt were calculated. In addition, insult burden was quantified as the proportion of GMT (%GMT) within predefined thresholds for each variable: ICP > 20 mmHg (according to our treatment protocol [18]), PRx > 0.2 (suggestive of cerebrovascular autoregulatory limit [35, 36]), CPP below/within/above 60–80 mmHg (considered ischemic/normal/potential hyperemia ranges, respectively [18]), and ΔCPPopt below/within/above ± 5 mmHg (indicating deviation from or adherence to the optimal CPP target as defined in the COGiTATE trial [37]).

### Visualization method

Three custom R scripts developed by one of the authors (A.H.) were used to visualize ICP-, PRx-, CPP-, and ΔCPPopt-related insults during the first seven days post-ictus in relation to functional outcome. First, a modified implementation [19, 38] of Güiza et al.’s methodology [39] generated heatmaps depicting the relationship between combinations of insult intensity and duration for each cerebral physiological variable and outcome (Supplemental Methods 1). Second, temporal variation in safe and hazardous ranges was assessed by calculating %GMT across multiple predefined intervals for each variable, correlating these values with outcome, and subdividing the first seven days into eight-hour segments, with data density plots showing data availability [19, 40] (Supplemental Methods 2). Third, potential interactions between PRx and ICP, CPP, and ΔCPPopt, respectively, were examined by computing %GMT meeting both threshold criteria, correlating these with outcome, and visualizing the results via heatmaps alongside corresponding data density plots [19, 41] (Supplemental Methods 3).

### Functional outcome

Short-term functional outcome was assessed at discharge from the NIC or neurointermediate unit using the Glasgow Outcome Scale at Discharge (GODS), ranging from 1 (death) to 5 (good recovery) [42]. For descriptive analyses, GODS was dichotomized as unfavorable vs. favorable outcome (GODS ≤ 3 vs. > 3) and mortality vs. survival (GODS 1 vs. 2–5) at NIC discharge.

### Statistical analysis

Continuous variables were presented as medians (interquartile range [IQR]) and categorical variables as numbers (percentage [%]). To evaluate risk factors of secondary insults, i.e., %GMT within/outside certain thresholds as defined above, Spearman’s rank correlation analyses were conducted for the variables in relation to demography (age), clinical (GCS M at NIC admission), and radiological (maximal sCH volume) injury severity. A similar analysis was done to explore the association of these physiological, secondary insults with outcome (GODS). Statistical significance was defined as p value < 0.05. All statistical analyses were performed in R, version 4.4.2 (R Core Team, R Foundation for Statistical Computing, Vienna, Austria).

## Results

### Demographic characteristics, admission, imaging, and treatment variables

In the total cohort of 94 patients (Table 1), the median (IQR) age was 68 years (60–73) with a predominance of male patients (62 vs. 38%). The median CCI score was 0 (0–1), and the majority had no antithrombotic therapy (62%). At NIC admission, the median GCS M score was 6 (4–6) with 6% of patients presenting with pupillary abnormality. Most bleedings were uni- rather than bilateral (70 vs. 30%) and the median maximal sCH volume was 26 mL (16–37). sCH evacuation was required in 78% of the cases, while the remaining 22% were managed with EVD treatment alone. ICP monitoring had a median duration of 7 days (4–12). Median NIC stay was 9 days (6–16). At NIC discharge, the median GODS was 3 (3–3), where 21 patients (22%) had favorable outcome and 5 patients (5%) were deceased.

**Table 1:** Demography, admission, imaging, and treatment variables

Variable	All
**Patients**, ***n (%)***	94 (100%)
*Demography*	
**Age**,** median (IQR) years**	68 (60–73)
**Sex (Male/Female)**, ***n (%)***	58/36 (62/38%)
**Charlson Comorbidity Index**,** median (IQR) score**	0 (0–1)
**Antithrombotic agents (none/AP/AC/Both AP and AC)**, ***n (%)***	58/15/19/2 (62/16/20/2%)
*Neurological function*	
**GCS M at NIC admission**,** median (IQR) score**	6 (4–6)
**Pupillary abnormality at NIC admission**, ***n (%)***	6 (6%)
*Radiology*	
**sCH localization (uni-/bilateral)**, ***n (%)***	66/28 (70/30%)
**Maximal sCH volume (mL)**,** median (IQR)**	26 (16–37)
*Treatment*	
**sCH evacuation**, ***n (%)***	73 (78%)
**ICP monitoring**,** median (IQR) days**	7 (4–12)
**NIC stay**,** median (IQR) days**	9 (6–16)
*Outcome*	
**GODS at NIC discharge**,** median (IQR)**	3 (3–3)
**Favorable outcome at NIC discharge**, ***n (%)***	21 (22%)
**Mortality at NIC discharge**, ***n (%)***	5 (5%)

*NIC*, Neurointensive Care; *Outcome at NIC discharge = GODS*, Glasgow Outcome Scale - Discharge; *Favorable outcome*, GODS > 3; *Mortality at NIC discharge*, GODS = 1; *CCI*, Charlson Comorbidity Index; *AC*, Anticoagulant; *AP*, Antiplatelet; *GCS M*, Glasgow Coma Scale Motor; *sCH*, spontaneous cerebellar hemorrhage; *ICP*, Intracranial Pressure; *IQR*, Interquartile Range; *N/A*, Not Applicable

### Cerebral physiological variables during the first seven days and predictors of secondary insults

The median values (%GMT) of cerebral physiological variables within/outside predefined thresholds during the first seven days post-injury are described in Supplementary Table 1. The median ICP was 7 mmHg (3–10), with a %GMT ICP > 20 mmHg of 0.36% (0.08–1.63). The median PRx was 0.19, while its %GMT > 0.2 was 49%. The median CPP was 96 mmHg (89–103), with a %GMT < 60, 60–80, and > 80 mmHg were 0.25% (0.12–0.76), 15% (4–27), and 83% (69–96), respectively. The median CPPopt was 93 mmHg (84–97), while the %GMT with ΔCPPopt insults < -5, -5 to 5, and > 5 mmHg were 29% (19–37), 27% (22–31), and 41% (30–49), respectively.

In Spearman’s rank correlation analysis of risk factors of secondary physiological insults (Supplementary Table 2), higher age was associated with lower %GMT ICP > 20 mmHg (*r* = -0.33, *p* = 0.002), lower %GMT ΔCPPopt < -5 mmHg (*r* = -0.21, *p* = 0.04), and higher %GMT ΔCPPopt > 5 mmHg (*r* = 0.22, *p* = 0.04). Higher GCS M at NIC admission correlated with higher %GMT CPP > 80 mmHg (*r* = 0.23, *p* = 0.03), while larger maximal sCH volume correlated with higher %GMT ΔCPPopt < -5 mmHg (*r* = 0.25, *p* = 0.02).

### Cerebral physiological variables in relation to functional outcome - Spearman’s rank correlation analysis and outcome heatmaps

In formal statistical tests, using Spearman’s rank correlation (Table 2) vs. the %GMT of within/outside predefined thresholds, a greater %GMT with CPP insults < 60 mmHg was associated with lower GODS (*r* = -0.25, *p* = 0.02). Conversely, a higher %GMT with CPP insult > 80 mmHg was associated with higher GODS (*r* = 0.25, *p* = 0.02). Increased %GMT with ΔCPPopt < -5 mmHg was associated with lower GODS outcome (*r* = -0.22, *p* = 0.04). There were no significant associations between GODS and %GMT with ICP insults > 20 mmHg, %GMT with PRx insults > 0.2, %GMT with CPP insults 60–80 mmHg, and %GMT with ΔCPPopt insults − 5 to 5 mmHg and > 5 mmHg.

**Table 2:** Predictors of functional outcome during seven days post-injury – Spearman’s rank correlation analysis

Variable	GODS at NIC discharge	
	*r*	*p* value
ICP (mmHg)	0.06	0.58
ICP > 20 mmHg (%GMT)	0.02	0.85
PRx, coefficient	-0.12	0.25
PRx > 0.2 (%GMT)	-0.12	0.26
CPP (mmHg)	0.19	0.07
CPP < 60 mmHg (%GMT)	-0.25	***0.02***
CPP 60–80 mmHg (%GMT)	-0.12	0.07
CPP > 80 mmHg (%GMT)	0.25	***0.02***
CPPopt (mmHg)	0.09	0.43
ΔCPPopt < -5 mmHg (%GMT)	-0.22	***0.04***
ΔCPPopt − 5 to 5 mmHg (%GMT)	0.06	0.55
ΔCPPopt > 5 mmHg (%GMT)	0.04	0.72

*NIC*, Neurointensive Care; *Outcome at NIC discharge = GODS*, Glasgow Outcome Scale - Discharge; *ICP*, Intracranial Pressure; *PRx*, Pressure Reactivity Index; *CPP*, Cerebral Perfusion Pressure; *CPPopt*, Optimal CPP; *ΔCPPopt*, CPP – Optimal CPP; *%GMT*, Percentage of Good Monitoring Time

p values in bold and italics indicate statistical significance

r = rho, Spearman rank correlation coefficient

**Fig. 1:**
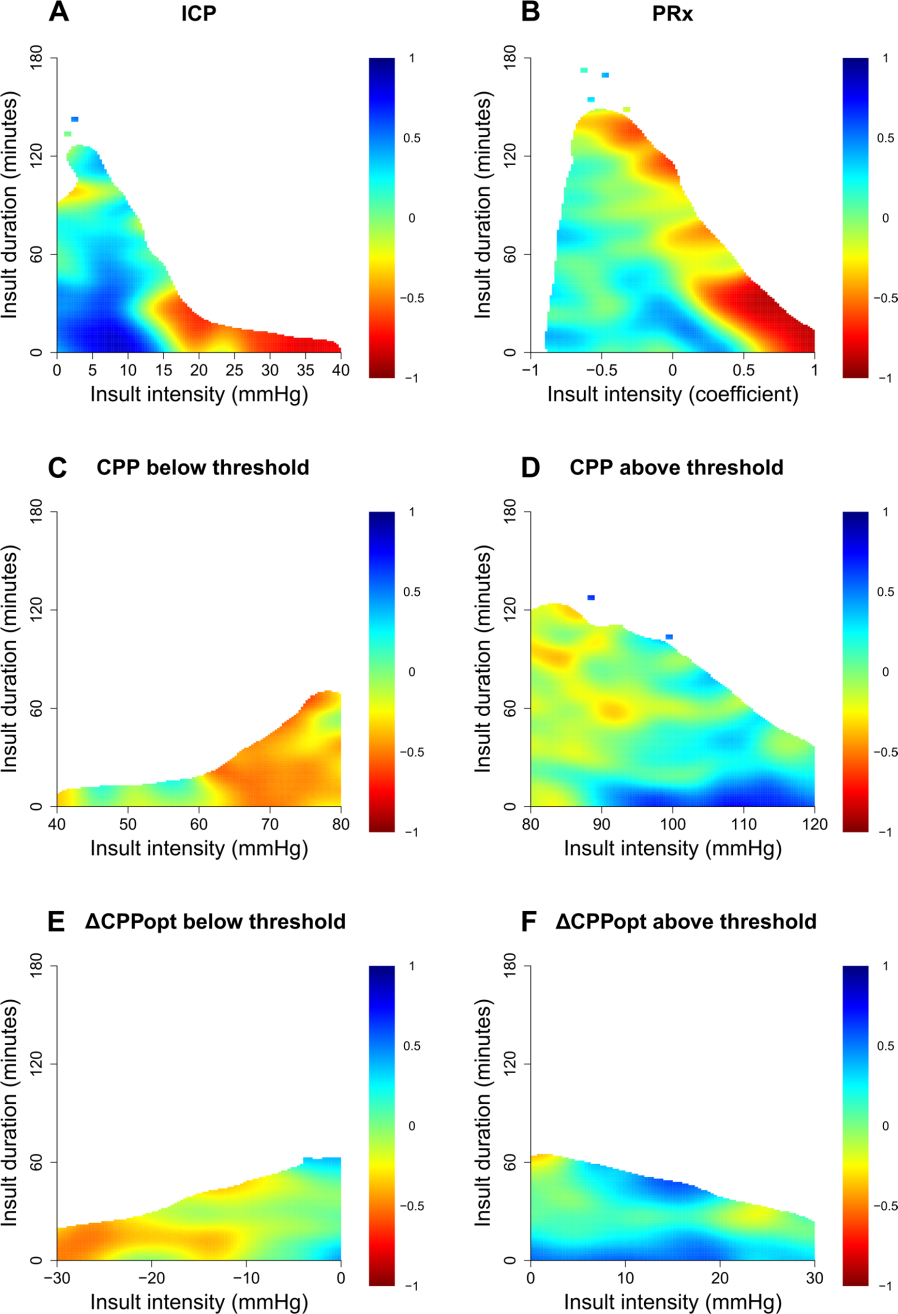
Visualization of insult intensity and duration of cerebral physiological variables in relation to functional outcome during the first seven days post-injury. The figure visualizes the combination of insult intensity and duration during the first seven days post-injury in relation to functional outcome for each cerebral physiological variable (**1A**-**1 F**). The jet color scale is used to visualize the correlation to favorable (higher GODS; blue) or unfavorable (lower GODS; red) outcome. Colored grid cells contain data for at least 20 patients with at least one insult, while cells containing less data were colored white. For ICP (**1A**), there was a transition towards unfavorable outcome for values > 15 mmHg regardless of the duration. For PRx (**1B**), there was a transition towards unfavorable outcome for insults > + 0.25 for durations of 40 min or longer, while durations of 60 min or longer expanded the unfavorable PRx insult from − 0.75 to + 0.50. For CPP < threshold (**1C**), an unfavorable zone was displayed for values between 60 and 80 mmHg regardless of the duration. For CPP > threshold (**1D**), no larger unfavorable zones were visible. For ΔCPPopt < threshold (**1E**), an unfavorable outcome zone was noted < -10 mmHg for durations of 20 min or shorter. For ΔCPPopt > threshold (**1F**), no larger unfavorable zones were noted. *CPP*, Cerebral Perfusion Pressure; *CPPopt*, Optimal CPP; *ΔCPPopt*, CPP – Optimal CPP; *GODS*, Glasgow Outcome Scale - Discharge; *ICP*, Intracranial Pressure; *PRx*, Pressure Reactivity Index

Outcome heatmaps were used to explore the relation between the cerebral physiological variables and GODS. In insult intensity and duration heatmaps, for ICP (Fig. 1A), a transition towards unfavorable outcome was observed for values > 15 mmHg regardless of the duration. For PRx (Fig. 1B), a transition towards lower GODS was visible for insults > + 0.25 for durations of 40 min or longer, while this transition occurred for shorter durations at higher intensities. For CPP < threshold (Fig. 1C), episodes below 80 mmHg were associated with worse outcome, regardless of the insult duration. Meanwhile, episodes of CPP > threshold (Fig. 1D) were, overall, associated with more favorable outcome. Lastly, episodes of ΔCPPopt < -10 mmHg (Fig. 1E) were linked to lower GODS, essentially for all durations, meanwhile ΔCPPopt > threshold (Fig. 1F) was associated with higher GODS. The corresponding data density visualizations of the combined insult intensity and durations are illustrated in Supplementary Fig. 2.

**Fig. 2:**
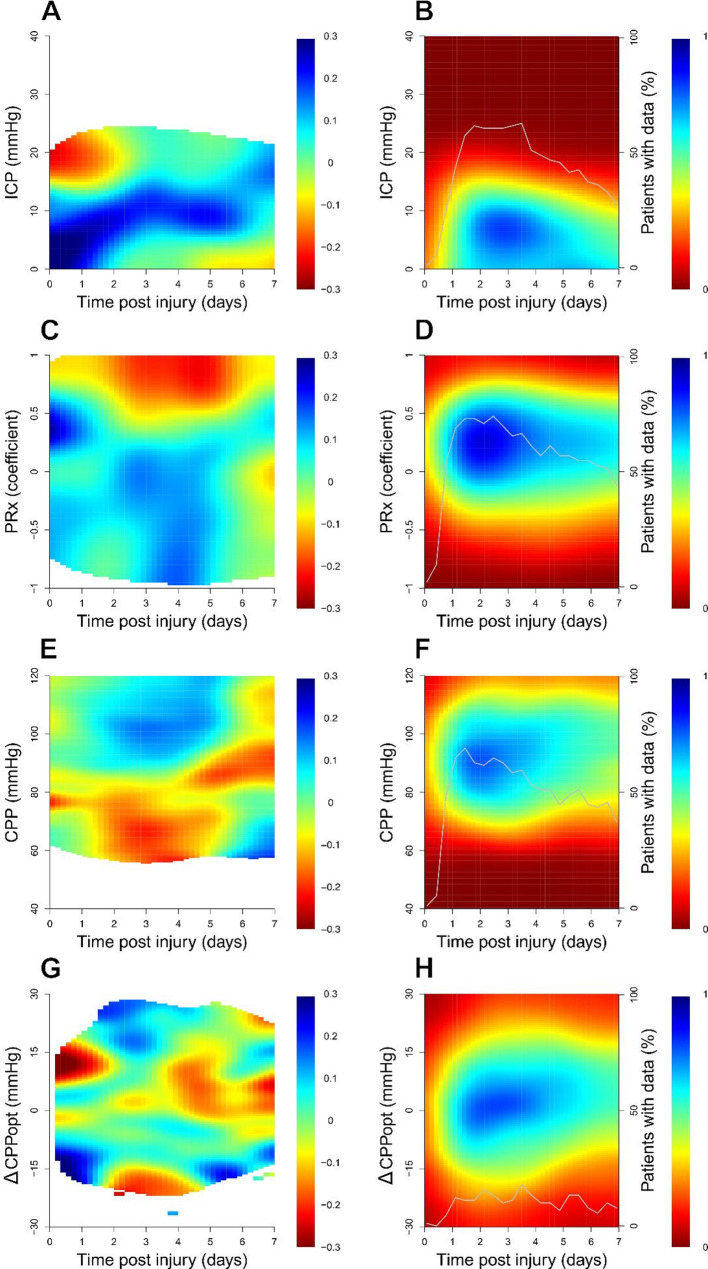
Visualization of cerebral physiological insults in relation to functional outcome during the first seven days post-injury. The figure illustrates the temporal dynamics of ICP-, PRx-, CPP-, and ΔCPPopt-insults during the first seven days post-injury in relation to functional outcome (**2 A**, **2 C**, **2E**, **2G**) and their respective data density (**2B**, **2D**, **2 F**, **2 H**). The jet color scale is used to visualize the correlation to favorable (higher GODS; blue) or unfavorable (lower GODS; red) outcome, and high (blue) or low (red) data density. Colored grid cells contain data for more than five patients with five minutes of monitoring time, while cells containing less data were colored white. For ICP (**2 A**), there was a clear trend where ICP values > 15 mmHg correlated to unfavorable outcome during the first two days of NIC. ICP ranged mainly from 0 to 15 mmHg during this period (2B). For PRx (2 C), a strong association with unfavorable outcome was observed for values between + 0.50 and + 1.00 during day 2–6, which condensed to values between + 0.75 to + 1.00 during day 0–1 and day 7. PRx primarily ranged from − 0.25 to + 0.75 during this period (**2D**). For CPP (**2E**), values < 80 mmHg were generally associated with unfavorable outcome during day 1–5, with this unfavorable range expanding from 80 to 100 mmHg day 6 and from 80 to 120 mmHg day 7, respectively. CPP mostly ranged from 70 to 115 mmHg during this period (**2 F**). For ΔCPPopt (**2G**), no larger red or blue zones were noted, as smaller zones were scattered throughout day 0–7. ΔCPPopt predominantly ranged from − 20 mmHg to 20 mmHg (**2 H**). *CPP*, Cerebral Perfusion Pressure; *CPPopt*, Optimal CPP; *ΔCPPopt*, CPP – Optimal CPP; *GODS*, Glasgow Outcome Scale - Discharge; *ICP*, Intracranial Pressure; *PRx*, Pressure Reactivity Index

To examine the temporal variation of the relationship between the %GMT of the cerebral physiological variables and functional outcome, heatmaps in eight-hour segments over the first seven days post-injury were created (Fig. 2). For ICP (Fig. 2A), there was a strong association between %GMT values > 15 mmHg and unfavorable outcome during the first two days, which gradually attenuated thereafter. ICP mostly ranged between 0 mmHg and 15 mmHg (Fig. 2B). For PRx (Fig. 2C), higher %GMT values > + 0.75 correlated with unfavorable outcome/lower GODS on the first day post-ictus, with the threshold expanding to > + 0.50 on subsequent days. PRx ranged mainly from − 0.25 to + 0.75 (Fig. 2D). For CPP (Fig. 2E), higher %GMT values below 80 mmHg correlated with lower GODS during day 1–5, with a slightly higher transition threshold during the later phase. CPP predominantly ranged from 70 mmHg to 115 mmHg (Fig. 2F). For ΔCPPopt (Fig. 2G), there was no clear-cut transition from favorable to unfavorable outcome of %GMT values over the first seven days. ΔCPPopt mostly ranged between − 20 mmHg and 20 mmHg (Fig. 2H).

**Fig. 3:**
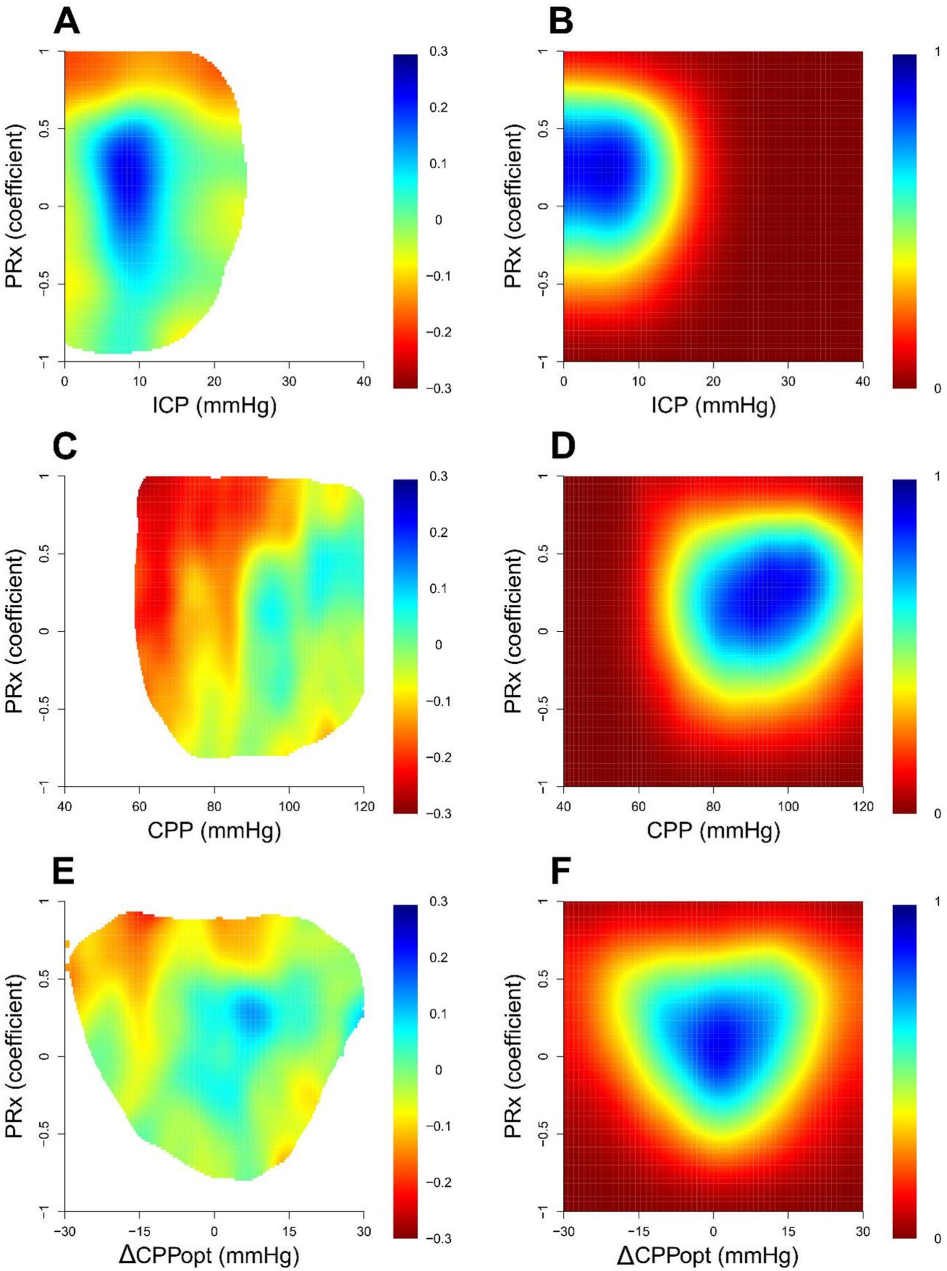
Visualization of the influence of PRx on the association of ICP, CPP, and ∆CPPopt in relation to functional outcome during the first seven days post-injury. The figure depicts the combination of specific PRx- and ICP-, CPP-, or ΔCPPopt-insults during the first seven days in relation to functional outcome. (**A**, **C**, **E**) and their respective data density (**B**, **D**, **F**). The jet color scale is used to visualize the correlation to favorable (higher GODS; blue) or unfavorable (lower GODS; red) outcome, and high (blue) or low (red) data density. Colored grid cells contain data for more than five patients with five minutes of monitoring time, while cells containing less data were colored white. For ICP (**3 A**), an unfavorable zone was noted for values from 0–25 mmHg with PRx > + 0.50. For CPP (**3 C**), lower values around 60 mmHg combined with PRx from − 0.25 to + 1.00 were unfavorable, whereas higher CPP around 100 mmHg combined with PRx + 0.50 to + 1.00 were unfavorable. For ΔCPPopt (**3E**), values < 15 mmHg combined with PRx around + 0.50 were unfavorable. The data density plots displayed the highest data frequency for PRx between − 0.50 and + 0.75 (B, D, F), for ICP from 0 to 15 mmHg (**3B**), for CPP from 70 to 120 mmHg (**3D**), and for ΔCPPopt from − 20 to 20 mmHg (**3 F**). *CPP*, Cerebral Perfusion Pressure; *CPPopt*, Optimal CPP; *ΔCPPopt*, CPP – Optimal CPP; *GODS*, Glasgow Outcome Scale - Discharge; *ICP*, Intracranial Pressure; *PRx*, Pressure Reactivity Index

To assess the interaction between autoregulatory status (PRx) and ICP, CPP, and ΔCPPopt in relation to functional outcome, combined insult visualizations were generated (Fig. 3). In the ICP/PRx visualization (Fig. 3A), a higher %GMT with PRx > + 0.50 was associated with lower GODS, largely independent of ICP levels across the range of 0–25 mmHg. In the CPP/PRx visualization (Fig. 3C), the combination of lower CPP and higher PRx was strongly associated with worse outcome. A transition toward lower GODS was observed at CPP values below 100 mmHg when PRx approached + 1.00, whereas this transition occurred at lower CPP levels, around 80 mmHg, when PRx values were closer to zero. In the ΔCPPopt/PRx visualization (Fig. 3E), a transition toward unfavorable outcome was observed for ΔCPPopt values below − 10 to -15 mmHg in the presence of PRx values exceeding + 0.50. Each plot’s data density is visualized in Fig. 3B, D, and F.

## Discussion

This study demonstrated a descriptive trend toward worse outcome for ICP values exceeding 15 mmHg. Positive PRx and sustained durations of such insults were associated with unfavorable outcome. CPP targets below 80 mmHg were associated with worse outcome. Moreover, PRx showed potential in refining optimal CPP ranges and negative ΔCPPopt was associated with worse outcome. These findings collectively suggest that sCH demands a distinct cerebral physiological monitoring and management framework, and that individualized, PRx-guided targets may be more appropriate than fixed population-level thresholds.

### ICP – low tolerance in the posterior fossa

Supratentorial measured ICP values were overall relatively low, and episodes of ICP elevation were managed with open EVD. Consistent with this, ICP demonstrated a weak association with functional outcome, and the transition toward unfavorable outcome occurred at lower pressure ranges (> 15 mmHg) than typically reported in other acute brain injuries such as supratentorial ICH, TBI, and SAH (> 20–22 mmHg) [4, 5, 18, 25]. Higher age was associated with lower %GMT ICP > 20 mmHg. This is likely because elder patients have more cerebral atrophy, which increases intracranial compliance and makes ICP rises less likely, and because clinicians may use less intensive monitoring and treatment in elder patients with poor prognosis, especially above 70–75 years. Furthermore, temporal aspects of ICP burden during the first two days post-injury were more strongly associated with unfavorable outcome than later insults. This suggests that an early ICP instability may reflect a vulnerable phase in sCH, where limited compensatory reserve and acute mass effect dominate clinical trajectories [1–3]. Later in the course, ICP may be more heavily influenced by treatment strategies, particularly CSF diversion, which may attenuate both absolute pressure levels and their association with outcome. Elevated supratentorial ICP in sCH may reflect obstructive hydrocephalus, a condition that is frequently amenable to treatment with open EVD [43]. This may partly explain why sustained high ICP values were uncommon and why duration-dependent ICP effects were less pronounced than in other acute brain injuries [18, 19]. However, supratentorial ICP may not fully capture infratentorial pressure dynamics. Previous work [1–3, 10, 11] has demonstrated different pressure gradients between supra- and infratentorial compartments, with posterior fossa structures exhibiting earlier compression and reduced tolerance to volume expansion despite relatively modest supratentorial ICP elevations. Such compartmental differences are most pronounced early after injury and tend to equilibrate over the subsequent days [10]. Taken together, supratentorial ICP may be unsuitable for infratentorial pathophysiology in sCH, however, early ICP elevations may serve as a marker of injury severity and limited physiological reserve. This reinforces the notion that extrapolation of supratentorial ICP thresholds to infratentorial pathology should be approached with caution, while also highlighting the importance of temporal context and treatment effects when interpreting outcome associations with ICP.

### PRx – a central determinant

Exploratory visualizations of insult intensity and duration, as well as timing demonstrated descriptive trends suggesting that sustained periods of impaired autoregulation were associated with unfavorable outcome. Particularly, PRx exceeding + 0.25 for prolonged durations, were linked to unfavorable outcome, with the strongest effects observed between day 2 and 6 post-injury. The temporal expansion of these unfavorable PRx zones suggests cumulative vulnerability as autoregulatory failure persists. PRx emerged as a central modifier of outcome associations with other cerebral physiological variables to some extent. These findings align closely with prior studies in supratentorial ICH, SAH, and TBI, where impaired PRx has been repeatedly associated with increased mortality and unfavorable functional outcome [18, 19, 22, 44]. In supratentorial ICH, impaired autoregulation has been shown to narrow the safe zones of ICP and CPP [18]. In SAH, disturbed autoregulation has been linked not only to global outcome but also delayed cerebral ischemia and infarct development [44]. Similarly, in TBI, PRx-guided management strategies have been proposed to individualize CPP targets and reduce secondary brain injury [37]. The present study suggests that autoregulatory failure is a shared pathophysiological mechanism of secondary brain injury across other acute brain injuries too, despite differences in anatomy and primary pathology. Notably, the expansion of unfavorable PRx zones over time suggests increasing vulnerability as autoregulatory failure persists. This observation supports the concept of dynamic interpretation of PRx, where the timing, duration, and interaction with ICP and CPP are critical determinants of secondary brain injury [18, 45]. Even though these trends exist, it is worth acknowledging that the association between PRx and outcome was not clear-cut as in other mentioned pathologies. This may in part be explained by treatment-related factors influencing intracranial compliance, including open EVD management and suboccipital bony decompression with duraplasty, which increase compliance and attenuate ICP pulse amplitudes, potentially reducing the reliability of PRx [46]. Following decompression and duraplasty, additional CSF diversion or leakage, including pseudomeningocele formation, may further lower ICP values and increase compliance [47]. Moreover, PRx represents a global measure of CPA, whereas infratentorial cerebral blood flow constitutes a relatively smaller component and may differ physiologically from supratentorial autoregulation, potentially limiting PRx validity in this context. However, supra- and infratentorial ICP differences typically equilibrate after a few days [10], short-term ICP variation, including global slow waves and ICP increase/decrease, are likely synchronous despite varying amplitudes making supratentorial PRx still relevant in capturing infratentorial autoregulation aspects. Consistently, a case series of patients with posterior fossa lesions demonstrated similar supra- and infratentorial PRx values [48]. However, further studies are warranted to draw definitive conclusions regarding supra- vs. infratentorial PRx. Nevertheless, in our study, PRx was associated with outcome, underscoring its potential relevance in sCH, while highlighting the importance of cautious interpretation in settings of altered compliance and posterior fossa pathology.

### CPP – time- and autoregulation-dependent effects

Lower CPP values (< 60–80 mmHg) showed an association with unfavorable outcome, while the threshold for favorable outcome was relatively high (> 80 mmHg). Furthermore, it seemed to have some temporal variance, as this threshold increased during the last observed days. Early vulnerability to low CPP mirrors findings from other acute brain injuries such as supratentorial ICH, SAH, and TBI, where early hypoperfusion has been implicated in secondary brain injury and unfavorable outcome depending on timing and autoregulatory status [49–51]. Interestingly, in the present visualization, CPP values < 80 mmHg were associated with unfavorable outcome during the first five days, despite current treatment protocols targeting CPP > 60 mmHg [5]. Specifically, treatment protocol suggests active treatment to raise CPP for values < 60 mmHg, but allows for fluctuations from 60 to 100–120 mmHg. It is important to keep in mind that CPP in this study is based on supratentorial ICP measurements, hence, the actual infratentorial CPP value is probably lower in case of elevated infratentorial ICP [52]. This raises the possibility that posterior fossa pathology imposes higher perfusion requirements during the acute phase, potentially due to altered perfusion requirements and/or brainstem vulnerability, which has also been noted in SAH, where early CPP augmentation is often required to maintain adequate cerebral blood flow despite normal or mildly elevated ICP [22, 25]. One possible contributor to this similarity between sCH, supratentorial ICH, and SAH may be the presence of pre-existing chronic vascular comorbidities among the cohorts – causing a right-shift in the autoregulation curve [5, 53]. Moreover, these findings suggest that strict ABP reduction may not be necessary at all stages, especially after the acute period when most hematoma expansion has stabilized. Furthermore, combined CPP-PRx analyses revealed that mainly low CPP values were associated with unfavorable outcome regardless of PRx value, however, in the presence of higher PRx values higher CPP levels were required to maintain favorable outcome. Low CPP combined with impaired PRx likely reflects ischemic vulnerability. Similarly, PRx has been shown to reflect the lower limit of autoregulation in TBI and SAH, characterized by high PRx at low CPP, while in TBI it may also indicate hyperemic states (which may be more common in this disease) at high CPP in the presence of elevated PRx [54, 55]. Our results of combined CPP-PRx analyses reinforce the concept that CPP should not be interpreted in isolation and, instead, analyzed with autoregulatory status.

### ΔCPPopt – diagnosis-specific limitations

In line with findings from other acute brain injuries including supratentorial ICH, TBI, and SAH [18, 19, 24, 56], our results indicate that CPP values below CPPopt are associated with worse outcome in sCH. While the temporal analysis over seven days yielded less distinct associations, this is likely attributable to the relatively low CPPopt yield and the limited amount of available data within 4-hour intervals. In contrast, analyses based on overall monitoring time consistently demonstrated CPP below CPPopt was associated with unfavorable outcome. Several methodological considerations may influence the performance of CPPopt in sCH, largely overlapping with those relevant for PRx, including reliability constraints related to PRx signal quality, the validity of a global autoregulatory metric in the setting of infratentorial pathology where the primary injury is located, and the inherently limited CPPopt yield [18, 55–57]. Taken together, these findings support the relevance of CPPopt in sCH, while emphasizing the need for cautious interpretation and contextual integration with PRx rather than reliance on CPPopt as an isolated target.

### Methodological considerations

The main strength of this study is that we included detailed clinical variables and high-frequency cerebral physiological data, which, to our knowledge, has not been previously researched upon. Advanced analytical approaches were used to assess the role of cerebral physiology in relation to functional outcome. Particularly, granular visualization methods of cerebral physiological insults and duration, as well as temporal dynamics on unfavorable and favorable outcome zones over the most acute phase post-injury (first week). Additionally, the interaction between the CPA status (PRx) on the tolerability of exceeding certain thresholds of other cerebral physiological variables was also investigated. Limitations must also be acknowledged. First, albeit this is the first study of its kind in sCH, the retrospective single-center design limits causal inference and generalizability. However, the use of continuous high-frequency cerebral physiological data, standardized NIC management protocols, and consistent outcome assessment strengthens internal validity. Second, outcome was assessed at NIC discharge, reflecting early neurological status rather than long-term functional recovery. While early outcome measures can be challenging to evaluate and are possibly too early for severe cases of sCH, it can still be valuable to examine since the effects of secondary insults without attenuation of all events post-NIC. Third, ICP was measured supratentorial, which may underestimate true infratentorial pressure dynamics [7, 10, 11]. This limitation is inherent to current clinical practice and underscores the challenge of monitoring posterior fossa physiology. Future studies are warranted to investigate infratentorial ICP monitoring and the potential utility of PRx derived from infratentorial measurements. Fourth, visualization-based analyses are exploratory by nature and should be interpreted as hypothesis-generating, and do not explicitly demonstrate the cause. Instead, such approaches may be particularly valuable in complex, dynamic systems, where traditional summary statistics fail to capture clinically relevant patterns. Additionally, they may reflect the underlying injury, local NIC thresholds, and treatments instead. Fifth, some cerebral physiological values were relatively rare as shown by the data density plots, and the outcome analyses in these regions should be interpreted cautiously. This is noted particularly for ICP above 20 mmHg, which was a rare event in this posterior fossa pathology. Hence, these few episodes may be caused by neurological wake-up tests or nursing. Sixth, in line with previous discussion, it is worth mentioning the influence of open EVD management and its effects on ICP and PRx. Therefore, it could be argued to exclude such episodes, however, this data is not available for the selected study period. Seventh, PRx is associated with a low signal-to-noise ratio, meaning the visualization plots have to some extent been influenced by noise. Eighth, data on pharmacological management of hemodynamic disturbances – including hypotension (vasopressor/intravenous fluid administration) and hypertension (antihypertensive agent use) – were not systematically available for the study period. As such, the influence of these interventions on the observed physiological association could not be accounted for and represents an area for future investigation. Ninth, although measures of intracranial compliance, such as the regression of amplitude on pressure (RAP) and ICP amplitude, may be of considerable interest in this disease, we refrained from these analyses due to concerns regarding the reliability of ICP pulse waveform-based metrics in the presence of an open EVD. Continuous CSF drainage artificially dampens pulse amplitude by lowering ICP, disrupting the amplitude–compliance relationship underlying the RAP index [58] and rendering such metrics unreliable. Future studies might consider assessing compliance-based metrics during temporary periods of closed EVD, when waveform morphology is better preserved, or exploring alternative monitoring modalities less dependent on pulse amplitude dynamics. Lastly, secondary brain injury is inherently dynamic, multifactorial, and non-linear, but the limited cohort size (*n* = 94) prevented us from conducting further statistical analyses to fully elucidate clinically meaningful relationships. Accordingly, the descriptive Spearman correlation analyses should be interpreted in this context, as the modest cohort size limits statistical power to detect weaker associations between cerebral physiological variables and outcome. Hence, prospective studies are warranted to validate these findings and also adjust for relevant confounders.

## Conclusions

Cerebral physiological monitoring in sCH provides valuable prognostic insights. For ICP, a descriptive trend toward worse outcome was observed when exceeding 15 mmHg, although, this did not reach statistical significance. Importantly, optimal CPP targets in sCH appear to differ from those established in supratentorial brain injuries such as TBI, where targets of 60–70 mmHg are commonly applied. In sCH, higher CPP values (> 80 mmHg) were associated with favorable outcome, which may reflect the frequent presence of pre-existing cerebrovascular comorbidity in this population, causing a right shift of the autoregulatory curve and thereby elevating the lower limit of adequate CPP. Furthermore, while PRx did not demonstrate a statistically significant association with outcome, exploratory heatmaps suggest that autoregulatory capacity may help delineate the individual lower CPP limit, supporting a role for PRx-guided, individualized CPP targets rather than fixed population-level thresholds. These findings should be interpreted as hypothesis-generating, hence, larger multi-center studies are warranted to validate sCH-specific physiological targets and clarify the clinical utility of autoregulation-guided management in this population.

## Supplementary Information

Below is the link to the electronic supplementary material.

Supplementary Material 1.

## Data Availability

The datasets used and/or analyzed during the current study are available from the corresponding author upon reasonable request.

## Abbreviations

ABP: Arterial Blood Pressure
CCI: Charlson Comorbidity Index
COGiTATE CPPopt: Guided Therapy: Assessment of Target Effectiveness
CPA: erebral Pressure Autoregulation
CPP: Cerebral Perfusion Pressure
CPPopt: Optimal Cerebral Perfusion Pressure
ΔCPPopt: Delta Optimal Cerebral Perfusion Pressure
CSF: Cerebrospinal Fluid
CT: Computed Tomography
EVD: External Ventricular Drain
GCS: Glasgow Coma Scale
GCS M: Glasgow Coma Scale Motor
GMT: Good Monitoring Time
%GMT: Percentage Good Monitoring Time
GODS: Glasgow Outcome Discharge Scale
ICH: Intracerebral Hemorrhage
ICP: Intracranial Pressure
IVH: Intraventricular Hemorrhage
NIC: Neurointensive Care
PRx: Pressure Reactivity Index
RAP: Regression of Amplitude on Pressure
SAH: Subarachnoid Hemorrhage
sCH: Spontaneous Cerebellar Hemorrhage
TBI: Traumatic Brain Injury
