# Supplementary material for: Temporal dynamics of ICP, PRx, CPP, and CPPopt in relation to functional outcome in spontaneous cerebellar hemorrhage
Source: Crit Care. 2026 Jun 10;30:301. doi: 10.1186/s13054-026-06136-0 (PMC13255323; doi:10.1186/s13054-026-06136-0)
Supplement: Supplementary file 1 — Supplementary Material 1. [file 13054_2026_6136_MOESM1_ESM.docx]

**Supplementary methods**

*Supplemental methods 1. Visualization of insult intensity and duration of cerebral physiological variables in relation to functional outcome during the first seven days post-injury*

The association between insult intensity and duration of ICP, PRx, CPP, and ∆CPPopt during the first seven days post-ictus in relation to outcome was explored in Figure 1. For each cerebral physiological variable, data was down-sampled to one-minute-resolution and short data gaps (≤ 10 minutes) were interpolated using linear approximation based on the nearest available values. This approach was primarily applied to reduce the exclusion of insult episodes caused by limited missing values. Insults were generally evaluated using above-threshold criteria; however, for CPP- and ∆CPPopt-insults, additional analyses using below-threshold definitions were performed to capture both cerebral hypo- and hyperperfusion and their potential association with outcome. The analyzed ICP range was 0-40 mmHg with a resolution of 1.00 mmHg, while PRx values ranged from -1.00 to +1.00 with a resolution of 0.05. CPP thresholds were 40-80 mmHg (below) and 80-120 mmHg (above), both using a resolution of 1.00 mmHg. ∆CPPopt thresholds were -30 to 0 mmHg (below) and 0 to 30 mmHg (above), also with a 1.00 mmHg resolution. For each insult category, the mean number of insults was correlated with GODS (1-5) using Spearman’s correlation test, with positive correlation coefficients indicating associations with favorable outcome. To attenuate high-frequency variability, Gaussian smoothing with a 2 SD kernel was applied. Correlation results were visualized using the jet color scale, where blue denoted association with favorable outcome and red indicated association with unfavorable outcome. Cells representing fewer than 20 patients experiencing at least one insult were colored white.

*Supplemental methods 2. Visualization of cerebral physiological insults in relation to functional outcome during the first seven days post-injury*

The relationship between %GMT spent within predefined ranges of ICP, PRx, CPP, and ∆CPPopt during the first seven days post-ictus in relation to outcome was explored in Figure 2. To characterize early temporal dynamics, each day was subdivided into consecutive eight-hour intervals representing the acute phase. The analyzed value ranges were 0-40 mmHg for ICP, 40-120 mmHg for CPP, and -30 to 30 mmHg for ΔCPPopt values, with a resolution of 1.00 mmHg, respectively. Meanwhile, PRx values ranged from -1.00 to 1.00, with a resolution of 0.05. For each physiological variable, a matrix was created in which columns corresponded to eight-hour time segments where within each segment %GMT was calculated and correlated with GODS (1-5) using Spearman’s correlation test. To reduce high-frequency variability, Gaussian smoothing of 2 SD kernel was applied. Outcome associations were visualized using the jet color scale, with blue indicating favorable outcome and red indicating unfavorable outcome. White cells represent missing data of instances in which fewer than five patients contributed at least five minutes of monitoring time. To further illustrate data availability, data density plots were created by normalizing the number of observations in each cell to the maximum count within the grid, yielding values between 0 to 1. These density maps were similarly smoothed and visualized using the jet color scale, where blue reflects high data density and red indicates low data density.

*Supplemental methods 3. Visualization of the influence of PRx on the association of ICP, CPP, and ∆CPPopt in relation to functional outcome during the first seven days post-injury*

The association between the combination of PRx with ICP, CPP, or ∆CPPopt, during the first seven days post-ictus and GODS was explored in Figure 3. PRx values ranging from -1.00 to +1.00 with a 0.05 resolution were combined with ICP (0-40 mmHg), CPP (range 40-120 mmHg), and ∆CPPopt (-30 to 30 mmHg), each evaluated at a resolution of 1.00 mmHg. This approach resulted in matrices comprising 1600 cells for the PRx-ICP combination and 3200 cells for the PRx-CPP combination and 2400 cells for the PRx-∆CPPopt combination. To enhance visual smoothness, each matrix was further subdivided into 3x3 subcells. For all variable combinations, the proportion of %GMT was computed for each cell and correlated with GODS (1-5) using Spearman’s correlation test. Gaussian smoothing with a 2 SD kernel was applied to reduce high-frequency variability. Associations with outcome were visualized using the jet color scale, with blue indicating favorable outcome and red denoting unfavorable outcome. Cells representing fewer than five patients contributing at least five minutes of monitoring time were displayed in white. To depict data availability, corresponding data density plots were created by normalizing cell counts to the maximum number of observations within each grid, yielding values between 0 and 1. Cells lacking data or surrounded by available grid cells with a combined weight of less than 0.1 were classified as unavailable and likewise colored white. Similar to the outcome heatmaps, Gaussian smoothing was applied to the density maps, which were visualized using the jet color scale, where blue reflects high data density and red indicates low data density.

**Supplementary table 1. Cerebral physiological variables during seven days post-injury – a univariate analysis**

| **Variable** | **All** |
| --- | --- |
| ICP (mmHg), median (IQR) | 7 (3-10) |
| ICP > 20 mmHg (%GMT), median (IQR) | 0.36 (0.08-1.63) |
| PRx, median (IQR) | 0.19 (0.11-0.29) |
| PRx > 0.2 (%GMT), median (IQR) | 49 (41-59) |
| CPP (mmHg), median (IQR) | 96 (89-103) |
| CPP < 60 mmHg (%GMT), median (IQR) | 0.25 (0.12-0.76) |
| CPP 60-80 mmHg (%GMT), median (IQR) | 15 (4-27) |
| CPP > 80 mmHg (%GMT), median (IQR) | 83 (69-96) |
| CPPopt (mmHg), median (IQR) | 93 (84-97) |
| ΔCPPopt < -5 mmHg (%GMT), median (IQR) | 29 (19-37) |
| ΔCPPopt -5 to 5 mmHg (%GMT), median (IQR) | 27 (22-31) |
| ΔCPPopt > 5 mmHg (%GMT), median (IQR) | 41 (30-49) |

*NIC,* Neurointensive Care*; Outcome at NIC discharge = GODS,* Glasgow Outcome Scale - Discharge*; Detrimental outcome,* GODS ≤ 3*; Beneficial outcome,* GODS > 3*; ICP*, Intracranial Pressure; *PRx*, Pressure Reactivity Index; *CPP*, Cerebral Perfusion Pressure; *CPPopt*, Optimal CPP; *ΔCPPopt*, CPP – Optimal CPP; *IQR*, Interquartile Range; *%GMT*, Percentage of Good Monitoring Time.

**Supplementary table 2. Predictors of secondary insult during seven days post-injury – Spearman’s rank correlation analysis**

|  | **%GMT ICP > 20 mmHg** | | **%GMT PRx > 0.2** | | **%GMT CPP < 60 mmHg** | | **%GMT CPP 60-80 mmHg** | | **%GMT CPP > 80 mmHg** | | **%GMT ΔCPPopt < -5 mmHg** | | **%GMT ΔCPPopt -5 to 5 mmHg** | | **%GMT ΔCPPopt > 5 mmHg** | |
| --- | --- | --- | --- | --- | --- | --- | --- | --- | --- | --- | --- | --- | --- | --- | --- | --- |
| **Variable** | ***r*** | **p value** | ***r*** | **p value** | ***r*** | **p value** | ***r*** | **p value** | ***r*** | **p value** | ***r*** | **p value** | ***r*** | **p value** | ***r*** | **p value** |
| Age | -0.33 | **0.002** | 0.04 | 0.70 | -0.03 | 0.81 | -0.001 | 0.99 | 0.04 | 0.69 | -0.21 | **0.04** | 0.02 | 0.89 | 0.22 | **0.04** |
| GCS M at NIC admission | 0.001 | 0.99 | 0.03 | 0.75 | -0.09 | 0.39 | -0.17 | 0.10 | 0.23 | **0.03** | -0.14 | 0.19 | 0.04 | 0.72 | -0.19 | 0.07 |
| Maximal sCH volume (mL) | -0.11 | 0.30 | 0.11 | 0.29 | 0.09 | 0.38 | 0.08 | 0.46 | -0.10 | 0.35 | 0.25 | **0.02** | 0.02 | 0.86 | 0.02 | 0.84 |

*NIC,* Neurointensive Care*;* *GCS M,* Glasgow Coma Scale Motor*; sCH,* spontaneous cerebellar hemorrhage*; ICP,* Intracranial Pressure; *PRx*, Pressure Reactivity Index; *CPP*, Cerebral Perfusion Pressure; *CPPopt*, Optimal CPP; *ΔCPPopt*, CPP – Optimal CPP; *%GMT*, Percentage of Good Monitoring Time.

p values in bold and italics indicate statistical significance.

r = rho, Spearman rank correlation coefficient.

**Supplementary figure 1. Flowchart of inclusion and exclusion of patients.**

**269** patients with spontaneous cerebellar hemorrhage treated at the NIC unit

**175** patients excluded:

100 <12 hours ICP monitoring

63 Due to secondary etiology

11 Due to missing data

1 <18 years

**94** patients included

The flowchart illustrates the patient inclusion of this study. Initially, 269 patients with sCH (diagnostic codes I61.3 and I61.4) treated at the NIC or neurointermediate unit, Uppsala University Hospital, between 1 January 2008 and 31 August 2024, were evaluated for eligibility. The evaluation resulted in exclusion of 175 patients; 100 patients with less than 12 hours of total ICP monitoring during the first seven days post-injury; 63 patients with a secondary etiology (e.g., traumatic hemorrhage, intracranial tumors, vascular malformations, or neurosurgical procedures), 11 patients with missing clinical and radiological data, and 1 patient younger than 18 years of age. Finally, 94 sCH patients were eligible for inclusion in this study.

**Supplementary figure 2. Data density heatmaps of insult intensity and duration of cerebral physiological variables in relation to functional outcome during the first seven days post-injury**

**
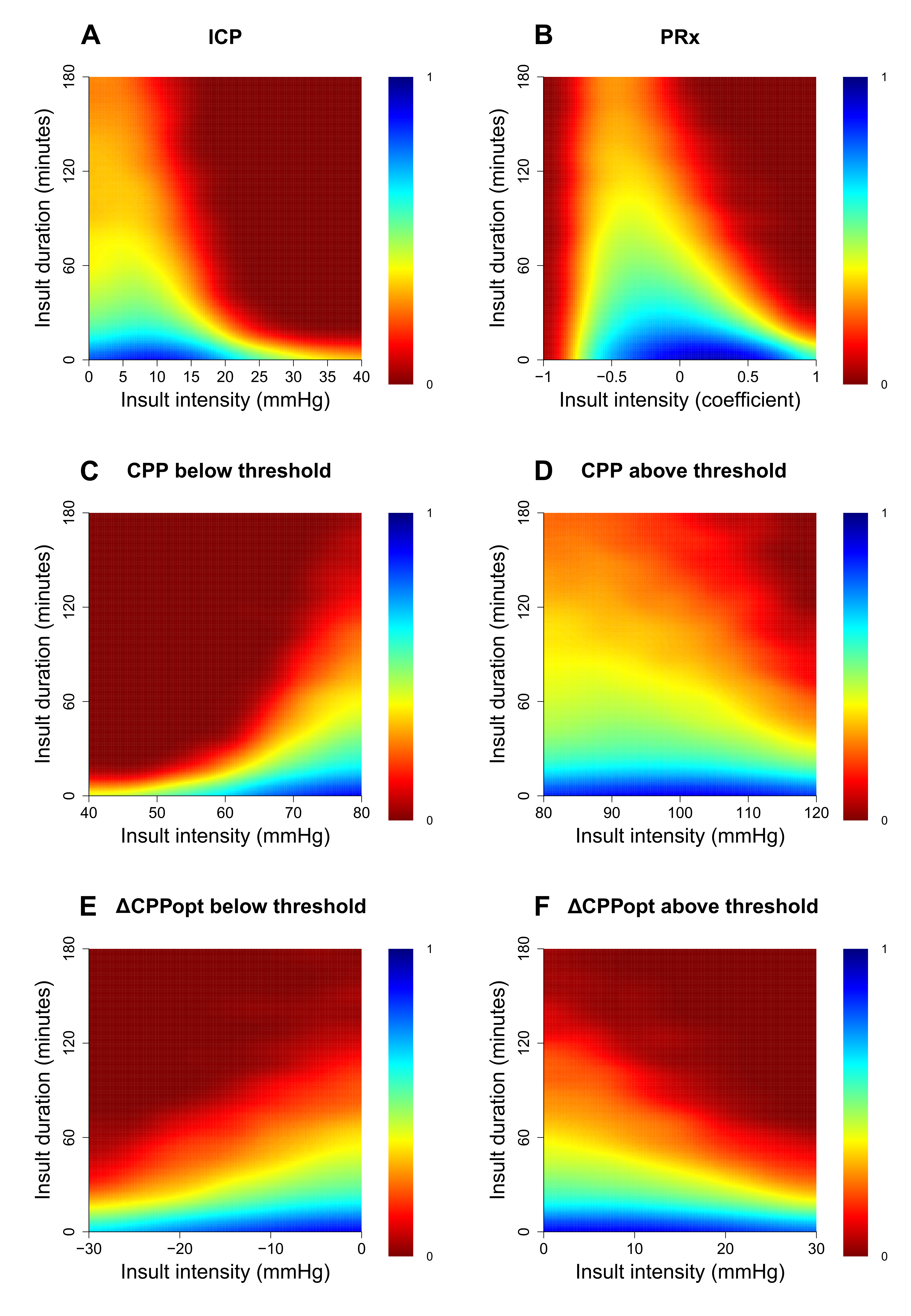
**

The figure visualizes logarithmic data density of the combination of insult intensity and duration during the first seven days post-injury in relation to functional outcome for each cerebral physiological variable (2A, 2B, 2C, 2D, 2E, 2F). The jet color scale is used to visualize the correlation with high data density (blue color) or low data density (red color). For ICP (2A), data density was highest for insults 0 to 20 mmHg for durations up to 60 minutes. For PRx (2B), data density was greatest for insults -0.50 to +0.80 for durations up to 115 minutes. For CPP < threshold (2C), data density was highest for intensities 60 to 80 mmHg and durations up to 60 minutes. For CPP > threshold (2D), data density was also highest for intensities 80 to 120 mmHg and durations up to 80 minutes. For ΔCPPopt < threshold (2E), data density was greatest at intensities -30 to 0 mmHg with durations up to 60 minutes. For ΔCPPopt > threshold (2F), data density was also greatest at intensities 0 to 30 mmHg with durations up to 60 minutes.
